# Evaluation of Cardiovascular Response to Isometric Handgrip Exercise in Obese Individuals

**DOI:** 10.7759/cureus.41898

**Published:** 2023-07-14

**Authors:** Kaushal Kishor Keshari, Tarun Kumar, Sunita LNU, Chandan Kumar, Manish Kumar

**Affiliations:** 1 Physiology, Indira Gandhi Institute of Medical Sciences, Patna, IND

**Keywords:** obese and non-obese patients, high systolic blood pressure, diastolic blood pressure, isometric, cardiovascular, cardiovascular assessment, isometric hand grip

## Abstract

Background

The variability in hemodynamic responses to isometric handgrip exercise in people with different body mass indices (BMIs) is noteworthy due to the frequent incidence of hypertension, obesity, and related cardiovascular illnesses in India. This investigation may be necessary to anticipate and prevent excessive heart strain during static activities. Therefore, this study aimed to evaluate the impact of isometric handgrip exercise on cardiorespiratory responses in an Indian population with varying BMI levels. The objective was to compare the cardiovascular responses of obese and non-obese individuals to an isometric handgrip exercise test (heart rate, systolic blood pressure, diastolic blood pressure, rate pressure product, and mean arterial pressure).

Methodology

This study was conducted from April 2021 to October 2022. Measurements were obtained using a pedestal-style weighing scale with a maximum capacity of 120 kg and an accuracy of 100 g at the Department of Physiology, Indira Gandhi Institute of Medical Sciences, Patna. Sphygmomanometer was used for measuring blood pressure, and a stopwatch was used to time the duration of each prolonged isometric contraction performed by the participants using a handgrip dynamometer. Baseline measurements of heart rate, blood pressure, cardiac output, and heart rate variability were done during the final three minutes of the rest phase and once more throughout the 10-minute recovery period that followed the isometric handgrip exercise.

Results

The average heart rate increased during the isometric handgrip exercise in the obese group (13.02 ± 1.88 beats per minute). Systolic blood pressure increased by 16.40 ± 2.65 mmHg and 23.66 ± 3.14 mmHg in the obese group and the normal weight group, respectively. The diastolic blood pressure increased similarly in the overweight and normal weight groups, measuring 18.64 ± 3.63 mmHg and 12.14 ± 1.95 mmHg, respectively. Furthermore, the mean blood pressure increased by 20.45 ± 3.13 mmHg and 13.67 ± 1.64 mmHg, respectively, both in the normal and overweight groups.

Conclusions

Obese individuals had greater resting heart rates, systolic blood pressure, and diastolic blood pressure than non-obese individuals. Following an isometric handgrip workout, non-obese individuals reported greater increases in heart rate, systolic blood pressure, and diastolic blood pressure than obese individuals.

## Introduction

A rise in non-communicable diseases such as cardiovascular disease (CVD) and fundamental changes in disease patterns have been brought about by the expansion of the population, growing urbanization, aging populations, and the spread of unhealthy lifestyles globally [1]. The World Health Organization (WHO) statistical analysis revealed that CVD is the primary reason for mortality worldwide [2]. Hypertensive disorder is an adverse condition for CVD. Hypertension, or the persistent rise of resting arterial blood pressure (BP), is managed by lifestyle modifications such as quitting smoking, decreasing weight, exercising, eating a nutritious diet, and ingesting less sodium; if these modifications are unsuccessful, antihypertensive medicines are added as treatment [3,4]. Increased physical activity and decreased idle time are critical techniques for managing and preventing hypertension. Undoubtedly, lowering BP remarkably reduces the likelihood of cardiovascular and cerebrovascular morbidity and mortality [3]. Similar to India, Indo-Asian countries struggle with the peculiar dual problems of the increasing number of overweight and obese individuals and a continuing undernutrition crisis. A rise in body weight affects cardiovascular health, depending on the age group [5]. Overweight people with low physical fitness have adverse effects on their vasculature, which puts them at higher risk for increased cardiovascular reactivity [3]. The body mass index (BMI), which also acts as a stand-alone adverse condition for cardiovascular morbidity and mortality, is crucial for calculating body weight. [4] Additionally, studies have shown a connection between BMI and cardiac autonomic nerve system modifications, which regulate the cardiovascular system both at rest and during physical activity [3-6]. It has been demonstrated that systemic exercise lowers the risk of cardiac disease by restoring normal BP and body weight. Exercise increases metabolic demands, which forces the body to modify its circulatory and respiratory systems. Additionally, exercise alters sympathetic and parasympathetic activity, cardiac output, total peripheral resistance, and mean arterial pressure (MAP). These adjustments depend on whether isometric or isotonic exercise is undertaken [3].

Isometric or static exercise is characterized by constant muscle contraction (i.e., an elevation in intensity) without a commensurate modification in the stretch of the affected muscle group. Recently, a meta-analysis indicated that isometric exercise training may result in greater average decreases in resting BP than cardiorespiratory exercise [4]. However, skeletal muscle is constricted during isotonic or dynamic exercise, changing the length of the muscle [6]. Handgrip exercise is an isometric training technique utilized in numerous studies to examine its effects on the cardiovascular system [7,8]. Literature grasped our attention to inquire into the effect of isometric handgrip exercise on cardiovascular responses in an Indian population with varying BMI to provide early diagnosis and treatment to halt the progression of CVDs. The variability in hemodynamic responses to isometric handgrip exercise in people with different BMIs is noteworthy due to the frequent incidence of hypertension, obesity, and related cardiovascular illnesses in India [9]. This investigation may be necessary to anticipate and prevent excessive heart strain during static activities.

Isometric exercise training, and particularly isometric handgrip training, may offer a beneficial new therapeutic adjuvant in the overall strategy for managing hypertension because they are widely applicable, affordable, and accessible to everyone globally. Even a small decrease in systemic arterial pressure and maintaining a healthy weight can significantly reduce morbidity and death in patients with cardiovascular risk factors [8]. Therefore, this study anticipated investigating the influence of isometric handgrip exercise on cardiorespiratory responses in an Indian population with varying BMI levels. The objective was to facilitate early detection and follow-up therapy to prevent the progression of CVDs. The findings of this research can serve as a guide for prescribing and implementing isometric exercise effectively in individuals with diverse BMI ranges, encompassing both healthy individuals and those with existing health conditions. The objective of this study was to compare the cardiovascular responses of obese and non-obese people to an isometric handgrip exercise test (heart rate, systolic blood pressure (SBP), diastolic blood pressure (DBP), rate pressure product (RPP), and MAP).

## Materials and methods

This cross-sectional study was conducted between April 2021 and October 2022 in the Department of Physiology, Indira Gandhi Institute of Medical Sciences (IGIMS), Patna. The institutional ethics committee (IEC) of IGIMS Patna granted ethical approval for the study protocol (approval number: 97/IEC/IGIMS/2021). Inclusion and exclusion criteria were developed to set specified standards for participant selection. The cohort for the study consisted of individuals who were selected based on their BMI and age range, specifically between 18 and 40 years. The participants were required to be non-drinkers, non-nicotine chewers, or nicotine addicts and needed to not exhibit any symptoms of severe or chronic illnesses. Only those who provided informed consent and were fully informed about the research objectives were included in the study. To prevent confounding variables associated with hypertension, participants with a history of acute or chronic diseases, as well as smokers, drinkers, and cigarette users, were removed from the study.

Sample size calculation

A total of 150 individuals (75 obese and 75 non-obese individuals) were chosen as the target sample size using the standard method for estimating sample size based on prevalence [10]. The following formula was used for calculating sample size: N = (Z)² × P(1 - P)/d². The prevalence was set at 50% because no appropriate statistics were available as no observational cohorts were available on the topic. With a 95% confidence level, the bound of error of 5% was chosen. The intended sample size of both groups was not met, so a sample of 100 was used instead (50 obese and 50 non-obese individuals).

Baseline measurements

Before enrolling in the study, the participants had their BP checked in a junior physician’s office at the IGIMS to determine whether or not pre-hypertension was present. The patients were subjected to three BP checks per appointment, performed at comparable times of the day on three different dates. After five minutes of rest, while seated, the BP in the left arm was measured with a sphygmomanometer (HEM 7120, OMRON Model), a BP cuff, and a stethoscope.

Measurement of body mass index

After completing the screening requirements, the individuals went to the lab on a different day to have their height and weight assessed. Quetlet’s index was used for measuring BMI [11]. A pedestal-style weighing scale (Crown, model: Crown Elite) with a maximum capacity of 120 kg and an accuracy of 100 g was utilized in this study for measuring BMI. The Stadiometer, equipped with a ruler and a horizontal headpiece, was adjusted on the participant’s head for height measurement. Based on their BMI, the participants were split into two groups. Patients classified as overweight had a BMI between 25 and 29.99 kg/m^2^, whereas the standard weight group and hereditary controls had a BMI between 18.5 and 24.99 kg/m^2^.

Measurement of blood pressure

Before testing on a different day after the screening, participants were asked to fast for three hours and desist from exercise for 12 hours. The individuals then participated in an isometric handgrip dynamometer test. Instructions on how to execute the isometric handgrip exercise test were given to each study participant. Before the exercise, they were allowed a 10-minute break. Individuals of both groups were then required to execute the isometric handgrip exercise under close guidance. Participants were told to tightly hold a Smedley handgrip spring dynamometer supplied by B D Instrumentation of India, squeeze the grips briefly, and then let go. By averaging three readings collected throughout the performance of the maximal handgrip contraction, the maximum isometric tension was determined. The individuals were then fitted with devices to track the physiological effects of BP, cardiac output, and heart rate variability.

Measurement of cardiovascular outputs

Non-invasive impedance cardiography equipment was used to measure cardiac output. Using a Biopac MP100 ECG data acquisition instrument, the heart rate was tracked beat-by-beat and represented by its reciprocal value (RR interval). Specialized heart rate variability software was used to evaluate heart rate variability in the temporal domain. The raw RR intervals were used to create a time domain metric known as the square root of the mean squared difference of successive RR intervals (RMSSD).

Isometric handgrip exercise

All patients were asked to sit quietly for five minutes. Baseline measurements of heart rate, BP, cardiac output, and heart rate variability were made during the final three minutes of the rest phase. Participants completed the isometric handgrip exercise for three minutes at 30% of their maximum isometric tension. Each participant was instructed to breathe normally during the test to reduce the chance of a Valsalva effect, and BP readings were taken through the arm that was not exercised. BP, heart rate, cardiac output, and heart rate variability were once more measured during the four-minute isometric handgrip exercise until exhaustion. The patient’s BP, heart rate, cardiac output, and heart rate variability were monitored throughout the 10-minute recovery period that followed the isometric handgrip exercise.

Statistical analysis

The gathered data were analyzed using SPSS version 23.0 (IBM Corp., Armonk, NY, USA). For the homogeneity and normality tests, Levene and Shapiro-Wilk tests were used. Contrary to categorical variables, shown as relative frequencies, continuous parameters were summarized as arithmetic mean ± SD for normally distributed data and median and interquartile range for non-normally distributed data. A comparison of baseline findings and clinical outcomes of both groups was done using the chi-square test for qualitative data and the Mann-Whitney U test for quantitative variables. A post-hoc pair-wise comparison using the Bonferroni correction for multiple comparisons was performed after generalized estimating equations were used to examine the impact of interventions on the cardiovascular parameters of the obese and non-obese groups. For measuring the mean difference, paired t-test was used. A p-value <0.05 was chosen as the significance level for each analysis.

## Results

The analysis used data from 100 different people in total. Out of the participants, 50 (50%) did not match the criteria for obesity, whereas the other 50 did (Table 1).

**Table 1:** Comparison of anthropometric parameters of the study groups. *: Statistically significant.

Variables	Normal weight	Overweight	P-value
Age (in years)	27.30 ± 5.95	25.84 ± 5.68	0.106
Height (in m)	1.65 ± 0.07	1.61 ± 0.05	0.008*
Weight (in kg)	62.45 ± 8.30	82.76 ± 7.16	<0.001*
Body mass index	22.77 ± 1.64	31.50 ± 1.22	<0.001*

The mean age of individuals in the normal weight group was 27.30 ± 5.95 years. In comparison, the mean age of individuals in the overweight group was 25.84 ± 5.68 years (p = 0.106).

The mean height of individuals in the normal weight group was 1.65 ± 0.07 m. The mean height of individuals in the overweight group was 1.61 ± 0.05 m (p = 0.008).

The average weight of individuals in the normal weight group was 62.45 ± 8.30 kg. The average weight of individuals in the overweight group was 82.76 ± 7.16 kg (p < 0.001).

The average BMI of individuals in the normal weight group was 22.77 ± 1.64 kg/m^2^, while the average BMI of individuals in the obese group was 31.50 ± 1.22 kg/m^2^ (p < 0.001).

In the overweight group (n = 50), the mean baseline pulse rate was 82.06 beats per minute (bpm), with a standard deviation of 6.17 bpm. Following the isometric handgrip exercise, their mean heart rate increased to 95.08 ± 5.27 bpm. In the non-obese group (n = 50), the baseline pulse rates ranged from 75.96 to 85.15 bpm. After the isometric handgrip exercise, their mean heart rate increased to 92.50 ± 4.98 bpm.

Regarding SBP, the average value for the obese group (n = 50) was 126.86 ± 6.63 mmHg at baseline. Following the isometric handgrip exercise, their average SBP increased to 143.26 ± 6.23 mmHg. In the non-obese group (n = 50), initially, the mean SBP was 114.72 ± 6.85 mmHg. After the isometric handgrip exercise, their mean SBP increased to 138.38 ± 7.46 mmHg.

For DBP, the average baseline value for the obese participants was 83.34 ± 3.87 mmHg. After the isometric handgrip exercise, their mean DBP increased to 95.48 ± 4.34 mmHg. Participants with normal weight had a mean DBP of 74.44 ± 4.62 mmHg at baseline. Following the isometric handgrip exercise, their mean DBP increased to 93.08 ± 4.14 mmHg.

Regarding MAP, the obese group (n = 50) had an average baseline MAP of 97.84 ± 4.22 mmHg. After the static handgrip activity, their mean MAP increased to 111.52 ± 4.18 mmHg. The non-obese group (n = 50) had a mean initial MAP of 87.86 ± 4.64 mmHg. Following the isometric handgrip exercise, their mean MAP increased to 108.32 ± 4.56 mmHg.

Regarding the RPP, the obese individuals (n = 50) had an average baseline RPP of 10,398.68 ± 798.67. Following the isometric handgrip exercise, their mean resting RPP increased to 13,613.10 ± 857.70. The non-obese individuals had an average baseline RPP of 8,722.66 ± 888.63. Their mean RPP increased to 12,825.86 ± 1,154.56 after the isometric handgrip exercise (Table 2).

**Table 2:** Comparison of the change in cardiovascular parameters during IHG exercise between the study groups. *: Statistically significant. DBP = diastolic blood pressure; SBP = systolic blood pressure; MAP = mean arterial pressure; RPP = rate pressure product; IHG = isometric handgrip; SD = standard deviation

Parameters	Non-obese (mean ± SD)	Obese (mean ± SD)	P-value (unpaired t-test)
Increase in DBP	18.64 ± 3.63	12.14 ± 1.95	<0.001*
Increase in SBP	23.66 ± 3.14	16.40 ± 2.65	<0.001*
Increase in MAP	20.45 ± 3.13	13.67 ± 1.64	<0.001*
Increase in RPP	4,103.20 ± 727.26	3,214.42 ± 3 44.92	<0.001*
Increase in pulse rate	16.54 ± 4.47	13.02 ± 1.88	<0.001*

In comparison to the normal weight group (16.54 ± 4.47 bpm), the average heart rate increased during the isometric handgrip exercise in the obese group (13.02 ± 1.88 bpm). SBP increased by 16.40 ± 2.65 mmHg and 23.66 ± 3.14 mmHg in the obese group and the normal weight group, respectively. The DBP increased similarly in the overweight and normal weight groups, measuring 18.64 ± 3.63 and 12.14 ± 1.95 mmHg, respectively. Furthermore, the mean BP increased by 20.45 ± 3.13 mmHg and 13.67 ± 1.64 mmHg, respectively, both in the normal and overweight groups.

## Discussion

Due to the rising prevalence of hypertension, obesity, and associated CVDs in India, it is imperative to examine the variations in hemodynamic responses to isometric handgrip exercise in individuals with varying BMIs [7]. It may help predict and stop the high cardiac burden during static tasks. The results of this study may provide a framework for prescribing and practicing isometric exercise to individuals with a range of BMIs who are both healthy and obese. Compared to people in the normal weight range, overweight people have a higher rate of cardiovascular death. Therefore, it was essential to evaluate the cardiovascular responses to isometric exercise in the Indian population with varying BMIs for early diagnosis and to provide additional therapy to stop the progression of CVDs. The objective of this study was to compare the cardiovascular responses of obese and non-obese people to an isometric handgrip exercise test (heart rate, SBP, DBP, RPP, and MAP). This study found that the average baseline pulse rate in the overweight group was 82.06 BPM, while the non-obese group had a range of 75.96 to 85.15 bpm. The isometric handgrip exercise increased the mean heart rate to 95.08 ± 5.27 bpm, while the average SBP increased to 143.26 ± 6.23 mmHg. The average baseline DBP increased to 95.48 ± 4.34 mmHg, while the average initial MAP for the non-obese group was 87.86 ± 4.64 mm Hg. The RPP for the obese group increased from 10,398.68 at baseline to 13,613.10 ± 857.70 after the isometric handgrip exercise. The non-obese participants’ RPP averaged 8722.66 ± 888.63, while the isometric handgrip exercise increased it to 12,825.86 ± 1,154.56.

An excessive buildup of body fat that puts people’s health in danger defines obesity [12]. This syndrome is linked to hemodynamic and metabolic abnormalities that are risk factors for hypertension, ischemic heart disease, and stroke, among other CVDs [13-15]. Given that some subgroups of people with idiopathic obesity may have altered autonomic nervous system function, the autonomic nervous system, which controls physiological processes, is assumed to be involved in the clinical symptoms associated with obesity [16-18]. Previous studies, such as by Laitinen et al. [19], have linked variations in autonomic activity to total body fat and central body adiposity. Detecting early sympathetic nerve dysfunction may be crucial for the treatment of obesity and its associated complications. This study utilized a handgrip dynamometer to investigate cardiovascular responses during continuous isometric hand grip exercises to assess autonomic function.

In this study, cardiac responses were observed in terms of pulse rate, SBP, DBP, and MAP. Three readings of each parameter were noted at one minute, 1.5 minutes, and two minutes; however, the average difference in elevation or reduction was noted in this study. The findings of this study revealed that overweight individuals had significantly higher resting heart rates, SBP, DBP, and RPP compared to overweight individuals (p < 0.05). In the obese group, the mean DBP increased to 12.14 ± 1.95 within two minutes of exercise. Meanwhile, the average increase in SBP was 16.40 ± 2.65 in the obese group. Interestingly, isometric exercise shows more increase in SBP and DBP. However, the readings of the obese group remained within physiological limits. These findings align with a study by Akhtar et al. [20], which reported higher mean values of resting SBP and DBP in the obese group after a prolonged isometric handgrip test. Increased sympathetic vasoconstrictor tone, elevated blood catecholamine levels, and insulinemia may contribute to these differences [20].

Because of the more significant circulatory load that obesity places on the heart due to higher BMI, obese people have higher baseline heart rates and BP. Sympathetic activation in obese individuals primarily stimulates renal neurons, leading to increased BP and salt and water retention [21]. The renin-angiotensin system, adipose tissue, renal sympathetic nerve activity, and increased blood volume are among the factors involved. High intrarenal pressure stimulates renal afferent neurons, activating renal mechanoreceptors and triggering the release of renin, angiotensin II, aldosterone, hyperinsulinemia, elevated fatty acid levels, and hyperleptinemia [21]. These processes contribute to an overactive sympathetic nervous system in obese patients, leading to elevated BP. However, individuals with normal weight reported a high mean increase in heart rates and BP after performing exercise for two minutes (p < 0.005).

Following an isometric handgrip exercise, both the normal and overweight groups in this study showed an elevated heart rate and an increase in SBP and DBP. However, compared to the obese group, the non-obese group showed a considerably more significant rise (p < 0.05). In examining the effects of obesity on the autonomic functioning of the heart in healthy individuals, Sharma et al. [22] obtained similar results. Their research showed that, compared to the non-obese group, the obese group experienced a minor increase in heart rate after isometric exercise. Following other studies, obese people differ less from average weight in their mean SBP and DBP readings following isometric handgrip exercise (p < 0.05). Intriguingly, DBP reduced in response to prolonged hand gripping, indicating that obese people have less sympathetic nerve activity than adults of average weight. Current findings are compatible with research by Akhtar et al. [20], which showed that obese people had less sympathetic autonomic function than non-obese controls. In addition, a study by Bedi et al. [23] utilizing the isometric handgrip technique showed that obese kids exhibited lower DBP than controls, which may indicate sympathetic insufficiency in these kids.

In their study, Rossi et al. [24] examined the autonomic changes in 23 obese patients (BMI = 37.2 ± 3.03 kg/m^2^) with 78 non-obese people (BMI = 22.5 ± 2.6 kg/m^2^). Their research revealed no statistically significant difference between the DBP responses of the two groups. According to Nageswari [25], overweight kids exhibit sympathetic cardiovascular dysfunction. Additionally, Valensi et al. [26] discovered a connection between sympathetic insufficiency and obesity, which is consistent with previous data. Ewing et al. [27] defined an increase in DBP in the prolonged handgrip test as abnormal if it was 10 mmHg or less, borderline if it was 11 to 15 mmHg, and normal if it was 15 mmHg or more. This hurried approach increases the risk of autonomic dysfunction in obese people. Heart rate and SBP are the two key factors that affect cardiac workload.

SBP and heart rate are added to create the RPP, which offers a precise, non-invasive, and simple-to-measure signal. It is strongly correlated with myocardial oxygen uptake and can be used to estimate the workload on the heart indirectly [28,29]. Cardiovascular events are more likely to occur when the RPP is 10,000 BPM or more, whereas an average RPP is less than 10,000 bpm [30,31]. In this study, a significant difference was observed in resting RPP among normal and overweight participants.

Similar findings were reported by Raut et al. [28], who found that obese women had considerably higher non-resting heart rate pressure values than non-obese women. This finding suggests higher myocardial oxygen consumption and potential heart hemodynamic stress increases. Obesity results in an increase in total blood volume and cardiac output because it puts metabolic processes under stress due to the body’s excessive weight. As a result, obese people have a higher cardiac burden at any given amount of effort [32]. Elevated SBP results from obese people’s increased stroke volume, which primarily accounts for their higher cardiac output. However, extra body fat throws the sympathovagal system’s delicate balance off, raising BP and the heart rate at rest. Obese patients have higher RPP, resulting in increased myocardial oxygen demand [33].

Study limitations

There are several limitations to this study. The study’s sample size is small, which may restrict how broadly the results can be applied. A greater sample size would provide a more accurate representation of the intended audience and boost the study’s statistical power. The study may have chosen people from a particular environment, demographic, or geography, which may not fairly represent the general population, raising the possibility of bias in participant selection and lowering the study’s external validity. The cross-sectional design used in this study only recorded data within a specified period of time. As a result, it is difficult to determine a causal link between obesity and the cardiovascular effects of isometric handgrip training.

## Conclusions

Obese individuals had greater resting heart rates, SBP, and DBP than non-obese individuals. Following an isometric handgrip workout, non-obese individuals reported greater increases in heart rate, SBP, and DBP than obese individuals. Therefore, non-obese persons responded to an autonomic function test better than obese people. In contrast to non-obese young adults, the current investigation found that obese young adults had reduced sympathetic autonomic function. Obese individuals have autonomic dysfunction, which is linked to cardiovascular issues. As a result, isometric handgrip training may help in the early diagnosis of autonomic dysfunction in obese individuals, hence, lowering the risk of various cardiovascular issues.

However, in this study, we used BMI to determine the prevalence of obesity. Utilizing biochemical criteria, such as measuring serum leptin, could have added more clarity to the evaluation of obesity. Additionally, it is important to consider the study’s bias and limitations.
